# Insulin levels and HOMA index are associated with exercise capacity in patients with type 2 diabetes and coronary artery disease

**DOI:** 10.1186/1758-5996-6-36

**Published:** 2014-03-10

**Authors:** Rune Byrkjeland, Elisabeth Edvardsen, Ida Unhammer Njerve, Harald Arnesen, Ingebjørg Seljeflot, Svein Solheim

**Affiliations:** 1 Center for Clinical Heart Research, Department of Cardiology, Oslo University Hospital Ulleval, PO box 4956, Nydalen N-0424, Oslo, Norway; 2 Center for Heart Failure Research, Oslo University Hospital, Oslo, Norway; 3 Faculty of Medicine, University of Oslo, Oslo, Norway; 4 Department of Pulmonary Medicine, Oslo University Hospital Ulleval, Oslo, Norway; 5 Norwegian School of Sport Sciences, Oslo, Norway

**Keywords:** Coronary artery disease, Type 2 diabetes, Insulin resistance, HOMA index, VO_2max_

## Abstract

**Background:**

Previous studies on type 2 diabetes have shown an association between exercise capacity and insulin resistance. In patients with coronary artery disease (CAD) exercise capacity is often reduced due to exercise-induced ischemia. We have investigated the association between glucometabolic control, including the homeostatic model assessment (HOMA) of insulin resistance, and exercise capacity in patients with type 2 diabetes and CAD with and without exercise-induced ischemia.

**Methods:**

In 137 patients (age 63.1 ± 7.9) cardiopulmonary exercise testing on treadmill was performed using a modified Balke protocol. The highest oxygen uptake (VO_2peak_) was reported as 30-s average. Fasting blood samples were drawn for determination of glucose, insulin and HbA1c. Insulin resistance (IR) was assessed by the HOMA2-IR computer model. Exercise-induced ischemia was defined as angina and/ or ST-depression in ECG ≥ 0.1 mV during the exercise test.

**Results:**

HOMA2-IR was inversely correlated to VO_2peak_ (r = -0.328, p < 0.001), still significant after adjusting for age, gender, smoking and BMI. Patients with HOMA2-IR above the median value (1.3) had an adjusted odds ratio of 3.26 (95 % CI 1.35 to 7.83, p = 0.008) for having VO_2peak_ below median (23.8 mL/kg/min). Insulin levels were inversely correlated to VO_2peak_ (r = -0.245, p = 0.010), also after adjusting for age and gender, but not after additional adjustment for BMI. The correlation between HOMA2-IR and VO_2peak_ was also significant in the subgroups with (n = 51) and without exercise-induced ischemia (n = 86), being numerically stronger in the group with ischemia (r = -0.430, p = 0.003 and r = -0.276, p = 0.014, respectively). Fasting glucose and HbA1c were not correlated with VO_2peak_ or AT.

**Conclusions:**

Insulin resistance, as estimated by fasting insulin and the HOMA index, was inversely associated with exercise capacity in patients with type 2 diabetes and CAD, the association being more pronounced in the subgroup with exercise-induced ischemia. These results indicate that insulin resistance is related to exercise capacity in type 2 diabetic patients with CAD, possibly even more so in patients with exercise-induced ischemia compared to those without.

## Background

Exercise capacity is an important prognostic factor in both healthy and diseased individuals [1-3]. Studies have shown reduced exercise capacity in patients with type 2 diabetes compared with non-diabetic subjects [4,5]. The reason for this reduction is not completely understood, but both cardiac and skeletal muscle dysfunction have been suggested to play a role in type 2 diabetes [6,7]. In patients with coronary artery disease (CAD), exercise capacity is often reduced, primarily because of myocardial ischemia [8]. Hyperglycaemia is related to both micro- and macrovascular complications, but there are conflicting results concerning the association between glucose control and exercise capacity [9-11]. Insulin resistance, on the other hand, has been found to be associated with reduced exercise capacity in patients with type 2 diabetes [11,12].

Because of the increasing prevalence of type 2 diabetes in the world and the elevated risk for CAD in patients with type 2 diabetes, there is a growing awareness of patients suffering both diseases [13,14]. These patients may have more compromised exercise performance than patients with either type 2 diabetes or CAD alone, and the limiting factors of their exercise capacity can be difficult to determine, especially as silent ischemia is a common condition in patients with type 2 diabetes and CAD [15]. Insulin resistance is present in most patients with type 2 diabetes and is associated with atherosclerosis [16], but the relationship between insulin resistance and exercise capacity in patients with both type 2 diabetes and CAD is not clarified.

The aim of the present study was to investigate the association between glucometabolic control, including HOMA index of insulin resistance, and exercise capacity in patients with type 2 diabetes and CAD with and without exercise-induced ischemia. Our hypothesis was that insulin resistance was inversely correlated with exercise capacity in type 2 diabetics with CAD, possibly influenced by the presence of exercise-induced ischemia.

## Methods

### Study population

The present study is part of an ongoing randomized, controlled, clinical trial investigating the effects of exercise training on progression of atherosclerosis and glucose control in patients with type 2 diabetes and CAD. Patients with type 2 diabetes and CAD (n = 137) were included at the Department of Cardiology, Oslo University Hospital, Ullevål, Oslo, Norway between August 2010 and March 2012. All study subjects had CAD verified with coronary angiography and known type 2 diabetes. Exclusion criteria were presence of severe diabetic complications like proliferative retinopathy and end stage renal disease, cancer, stroke or acute myocardial infarction within the last three months, unstable angina, uncompensated heart failure, serious arrhythmia, serious valvular disease, serious rheumatologic disease, chronic obstructive pulmonary disease stadium GOLD IV, thromboembolic disease, ongoing infections, serious musculoskeletal disorders and other disabilities seriously limiting the ability for physical activity. All patients gave informed, written consent to participate in the study. The study was approved by The Regional Ethics Committee, was conducted according to the Declaration of Helsinki and is registered at http://www.clinicaltrials.gov, NCT01232608.

### Cardiopulmonary exercise test

Established guidelines for exercise testing of cardiac patients were followed [17]. The cardiopulmonary exercise test was performed by walking on a treadmill using a modified Balke protocol [18]. After three minutes of warm up at 2.8, 3.8 or 4.8 km/hr, the inclination was increased by 2% each 60s from 4% at start to a maximum of 20%. If the participant was still able to continue, the speed then rose by 0.5 km/hr each minute until exhaustion, or ended by the physician for safety reasons. Gas exchange and ventilatory variables were continuously measured breath by breath by breathing into a Hans Rudolph two-way breathing mask (2700 series; Hans Rudolph Inc, Kansas City, USA). The breathing mask was connected to a metabolic cart (Vmax SensorMedics, Yorba Linda, USA) to assess the ventilation and the oxygen and carbon dioxide content of expired air. The highest oxygen uptake (VO_2peak_) was reported as the highest consecutive 30s average of oxygen uptake during the test. Maximal respiratory exchange ratio (RER) was registered. The rating of perceived exertion was obtained using the Borg scale (6–20) [19]. A capillary blood lactate sample was taken during seated rest 1–3 min after termination of the exercise test using haemolysed blood (ABL 700 series, Radiometer, Copenhagen, Denmark). Heart rate and ST-deviations were recorded continuously from the 12-lead ECG record (Cardiosoft, GE Marquette Medical Systems, Milwaukee, USA). ST-depressions ≥ 0.1 mV 60 ms after J-point with a ST-slope < 1 mV/s were defined as significant. Exercise-induced ischemia in this study was defined as significant ST-depression and/or development of typical angina symptoms during test. The blood pressure was automated recorded each second minute of the exercise test, immediately after completion and after two and four minutes during recovery (SunTech Tango^+^ Stress BP, SunTech Medical, Inc. Morrisville, USA). The anaerobic threshold (AT) was calculated by the ventilatory equivalent method and was determined by two independent investigators [8]. In cases of disagreement, a third investigator was consulted. If two investigators were not able to determine the AT, the variable was excluded.

### Laboratory measures

Blood samples were drawn by standard venipuncture between 0800 and 1000 AM after overnight fast and without medication taken since the preceding evening for determination of glucose, insulin, c-peptide and HbA1c. Insulin and c-peptide were measured by Delfia method (Perkin Elmer) and HbA1c by turbidimetric inhibition immunoassay (Roche). Glucometabolic control was measured by fasting serum levels of glucose and insulin, and fasting blood levels of HbA1c. Insulin resistance was assessed by the homeostatic model assessment, both the original HOMA-IR model [20] and the updated computer model HOMA2-IR [21,22]. Patients on insulin treatment were included in the calculations as glucose and insulin were believed to be in a steady state condition at the time of blood sample collection [23]. All HOMA2-IR values were calculated by entering glucose and insulin values in the computer model (not c-peptide). Urine samples from the same morning were collected for determination of albumin/creatinine ratio.

### Statistical analysis

Demographic data are given as proportions, mean (±SD) or medians (25, 75 percentiles) with skewed data. Correlation analyses were performed using Spearman rank test and trend analysis by Pearson Chi square test. Patients on insulin treatment were excluded from correlation analyses between serum insulin and VO_2peak_/AT. Multiple linear regression analyses were used to adjust for covariates. Age and gender were considered relevant covariates in all calculations. Additional covariates were obtained by investigating differences in baseline characteristics in dichotomized groups of both dependent and independent variables (p < 0.2). Each of the possible covariates was clinically evaluated whether they most likely represented true confounders or variables related to the dependent variable through common mechanistic pathways as the independent variable. In case of the latter, the variables were not adjusted for. BMI was considered a confounder, but as adiposity is closely related to insulin resistance and their associations with exercise capacity may share common features, multiple regression analyses were performed both with and without BMI as part of the relevant covariates. Values of insulin and HOMA2-IR were log transformed before entered in the regression models because of skewed data. Logistic regression was used to calculate odds ratio on dichotomized values. Comparisons between ischemic and non-ischemic groups were calculated by independent samples T-test, Chi-square test or Mann–Whitney test as appropriate. Statistical calculations were performed using SPSS version 18.0. P-value < 0.05 was defined as statistically significant.

## Results

Baseline characteristics of the study population are presented in Table 1, and their physical performances in Table 2. Fifty-one patients (37%) developed exercise-induced ischemia during test as defined in this study. Twelve patients terminated the test because of chest pain and/or dyspnoea, and the physician terminated three of the tests for safety reasons (large ST-depressions). One hundred and twenty-seven patients (93%) reached RER ≥ 1.10 and/or Borg scale (6–20) ≥ 17 during the exercise test.

**Table 1:** Baseline characteristics of the study population (n = 137)

**Characteristics**	
Age (years)	63.1 ± 7.9
Sex (male/female)	115/ 22
Ethnicity (Caucasian/non-Caucasian)	114/ 23
BMI (kg/m^2^)	29.2 ± 5.0
Waist circumference (cm)	106 ± 13
Systolic blood pressure (mmHg)	139 ± 17
Diastolic blood pressure (mmHg)	79 ± 9
Metabolic syndrome^¤^	105 (77%)
Albuminuria (incl. micro-)	34 (25%)
Current smoker	23 (17%)
Previous myocardial infarction	62 (45%)
Hypertension	100 (73%)
Years of diabetes*	9 (5, 15)
Creatinine (μmol/L)*	79 (68, 91)
LDL (mmol/L)*	2.0 (1.6, 2.6)
HDL (mmol/L)*	1.12 (0.94, 1.38)
Triglycerides (mmol/L)*	1.42 (1.06, 1.91)
HbA1c (%)*	7.4 (6.8, 8.3)
Glucose (mmol/L)*	8.1 (6.9, 9.8)
Insulin (pmol/L)*	57 (33, 102)
C-peptide (pmol/L)*	965 (713, 1290)
HOMA2-IR*	1.3 (0.7, 2.1)
Anti-platelet therapy	129 (94%)
Statin	128 (93%)
Beta-blocker	106 (77%)
ACE-inhibitor/ARB	96 (70%)
Metformin	101 (74%)
Sulfonylurea	47 (34%)
Gliptin	17 (12%)
Insulin/insulin-analogue	26 (19%)

Data presented as proportions or mean value ± SD if not otherwise stated.

ACE; angiotensin converting enzyme. ARB; angiotensin receptor blocker.

*Median (25, 75 percentiles). ^¤^International Diabetes Federation, 2006.

**Table 2:** Physical performance of the participants in the total population

**Cardiopulmonary exercise test**	
Exercise time (min:sec)	08:23 ± 02:39
Maximal RER*	1.17 (1.08, 1.23)
Blood lactate (mmol/L)*	6.3 (5.5, 8.4)
Maximal Borg rating scale (6–20)*	17 (17, 19)
Maximal heart rate (beat/ min)	139 ± 19
Maximal systolic blood pressure (mmHg)	188 ± 33
Maximal diastolic blood pressure (mmHg)	82 ± 14
VO_2peak_ (L/min)	2.17 ± 0.56
VO_2peak_ (mL/kg/min)	24.7 ± 5.9
AT (L/min)	1.68 ± 0.42
AT (mL/kg/min)	18.9 ± 4.1

Data presented as proportions or mean value ± SD if not otherwise stated.

RER; respiratory exchange ratio. AT; anaerobic threshold.

*Median (25, 75 percentiles).

HOMA2-IR was inversely correlated with both VO_2peak_ and AT (Table 3), and the correlation with VO_2peak_ was still significant after adjusting for age, gender, current smoking and BMI (Table 4).

**Table 3:** Correlation between measures of exercise capacity and glycemic control/insulin resistance

	**HbA1c(%)**	**Glucose (mmol/L)**	**Insulin**^**$ **^**(pmol/L)**	**HOMA2-IR**
VO_2peak_ (mL/kg/min)	r = -0.135	r = -0.140	r = -0.245	r = -0.328
	p = 0.117	p = 0.106	**p = 0.010**	**p < 0.001**
AT (mL/kg/min)	r = -0.141	r = -0.123	r = -0.225	r = -0.200
	p = 0.144	p = 0.208	**p = 0.036**	**p = 0.048**

^$^ Patients on insulin treatment excluded. AT; anaerobic threshold.

**Table 4:** Multiple regression analysis of association between insulin resistance and exercise capacity

	**logInsulin**^**$**^		**logHOMA2-IR**	
VO_2peak_ (mL/kg/min)	β = -0.261^a^	β = -0.111^b^	β = -0.326^c^	β = -0.207^d^
	**p = 0.005**	p = 0.293	**p < 0.001**	**p = 0.027**
AT (mL/kg/min)	β = -0.277^a^	β = -0.152^b^	β = -0.278^a^	β = -0.160^b^
	**p = 0.012**	p = 0.233	**p = 0.007**	p = 0.167

VO_2peak_ and AT as dependent variable, and insulin and HOMA2-IR as independent variable. Adjusted for ^a^age and gender, ^b^age, gender and BMI, ^c^age, gender and current smoking, ^d^age, gender, current smoking and BMI.

^$^Patients on insulin treatment excluded. β; Standardized beta coefficients.

**Figure 1:**
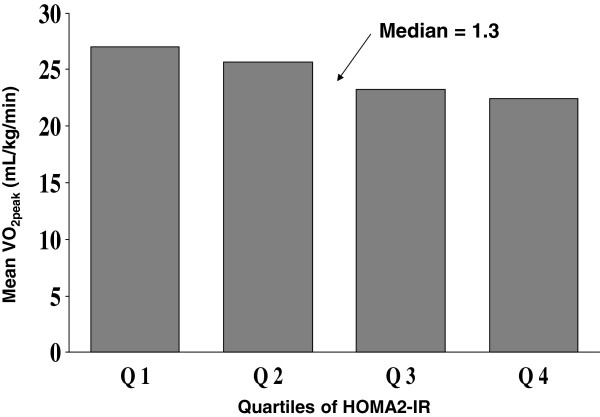
Peak oxygen uptake in quartiles of HOMA2-IR.

Trend analysis through quartiles of HOMA2-IR showed a significant inverse relationship between increasing HOMA2-IR and VO_2peak_ (p < 0.001) (Figure 1). Patients with HOMA2-IR above the median value (1.3) had an odds ratio of 3.26 (95% CI 1.35 to 7.83, p = 0.008) for having VO_2peak_ below median (23.8 mL/kg/min) after adjusting for age, gender, current smoking and BMI. There were no differences in medication in the quartiles of HOMA2-IR, except for use of insulin treatment, which was more frequent in the highest quartile compared with the three lower quartiles (p < 0.001). Replacing HOMA2-IR with HOMA1-IR did not change the results significantly. The levels of significance were, however, overall marginally stronger with the HOMA2-IR model compared to the HOMA1-IR model (data not shown).

Insulin levels were inversely correlated with VO_2peak_ and AT (Table 3), also after adjusting for age and gender, but not after additional adjustment for BMI (Table 4).

The ischemic and non-ischemic groups did not differ in baseline characteristics and physical performances, except for proportions of previous acute myocardial infarction (AMI) (31% vs 54%, respectively, p = 0.019) and Borg rating scale (6–20) during the exercise test (17 vs 18, p = 0.043). The correlation between HOMA2-IR and VO_2peak_ was statistically significant in both subgroups, being numerically stronger in the ischemic group (Table 5). Adjusting for age, gender and current smoking maintained the statistical significance in both groups, whereas it disappeared after additional adjustments for BMI (Table 6).

**Table 5:** **Correlations between VO**_**2peak **_**and glycemic control/ insulin resistance with (Panel A) (n=51) and without (Panel B) (n=86) exercise-induced ischemia**

**Panel A**	**HbA1c (%)**	**Glucose (mmol/L)**	**Insulin**^**$ **^**(pmol/L)**	**HOMA2-IR**
VO_2peak_ (mL/kg/min)	r = -0.026	r = -0.059	r = -0.339	r = -0.430
	p = 0.858	p = 0.683	**p = 0.021**	**p = 0.003**
**Panel B**				
VO_2peak_ (mL/kg/min)	r = -0.172	r = -0.180	r = -0.181	r = -0.276
	p = 0.116	p = 0.102	p = 0.155	**p = 0.014**

^$^ Patients on insulin treatment excluded.

**Table 6:** Multiple regression analyses of association between insulin resistance and exercise capacity in ischemic (Panel A) and non-ischemic (Panel B) patients

**Panel A**	**logInsulin**^**$**^		**logHOMA2-IR**	
VO_2peak_ (mL/kg/min)	β = -0.369^a^	β = -0.190^b^	β = -0.432^a^	β = -0.315^b^
	**p = 0.021**	p = 0.325	**p = 0.003**	p = 0.079
**Panel B**				
VO_2peak_ (mL/kg/min)	β = -0.205^a^	β = -0.030^b^	β = -0.289^c^	β = -0.180^d^
	p = 0.075	p = 0.810	**p = 0.005**	p = 0.101

VO_2peak_ as dependent variable, and insulin and HOMA2-IR as independent variables. Adjusted for ^a^age and gender, ^b^age, gender and BMI, ^c^age, gender and current smoking, ^d^age, gender, current smoking and BMI.

^$^ Patients on insulin treatment excluded.

Glucose control as measured by HbA1c and fasting glucose did not correlate with VO_2peak_ or AT, neither in the study population as a whole (Table 3), nor in the ischemic or non-ischemic subgroups (Table 5).

## Discussion

In the present study, our main finding was an inverse association between estimates of insulin resistance and exercise capacity in patients with both type 2 diabetes and CAD. The association was most pronounced between HOMA2-IR and VO_2peak_, but present also between HOMA2-IR and AT, and between insulin and both VO_2peak_ and AT. Further analysis showed that the inverse relationship between VO_2peak_ and HOMA2-IR was present also in the subgroups having ischemia or not, being numerically stronger in the ischemic group. Fasting glucose and HbA1c were not correlated with AT or VO_2peak_.

After adjusting for BMI, the correlation between HOMA2-IR and VO_2peak_ was still significant, while the correlations between HOMA2-IR and AT, and between insulin and both AT and VO2_peak_, were not. BMI, as a measure of adiposity, is of importance when considering the relationship between insulin resistance and exercise capacity as body fat may influence insulin resistance and also maximal oxygen uptake when measured as mL/kg/min. On the other hand, adiposity and insulin resistance are part of the metabolic syndrome [24] and may share patophysiological characteristics, and adjusting for BMI may result in an underestimation of the relationship between insulin resistance and exercise capacity. Nevertheless, as HOMA2-IR and VO2_peak_ were still significantly correlated also after adjusting for BMI, an association between insulin resistance and exercise capacity independent of BMI is indicated in these patients.

Our main result are in agreement with previous studies showing inverse association between insulin resistance and exercise capacity both in healthy subjects, relatives of patients with type 2 diabetes and in patients with type 2 diabetes [11,25]. There are also indications that treatment directed against insulin resistance may increase exercise capacity [26]. In type 2 diabetes, several mechanisms have been proposed by which insulin resistance and exercise capacity are related. Associations between insulin resistance and systolic and diastolic myocardial dysfunction, skeletal muscle dysfunction, attenuated mitochondrial activity and endothelial dysfunction have been reported [7,27-29]. All these pathological conditions can be related to impaired oxygen uptake and consumption.

The new finding in our study was that the association between insulin resistance and exercise capacity was also present in type 2 diabetes patients with CAD, indicating that insulin resistance may still influence exercise capacity even when myocardial function may be impaired by the presence of CAD. As exercise-induced ischemia in patients with CAD may have negative impact on exercise capacity, it could have been suspected that this association was weaker or non-existing in patients who developed ischemia. However, the subgroup analyses in our study showed a numerically stronger correlation among the patients with exercise-induced ischemia compared to those without, indicating that the negative association between insulin resistance and exercise capacity might be even stronger in type 2 diabetic patients with exercise-induced ischemia. These results should be interpreted with caution and only be considered as hypothesis generating. Nevertheless, they may by supported by the theory that insulin resistance may influence exercise capacity through pathways involving endothelial dysfunction and perfusion disturbances [11]. Insulin resistance is closely related to endothelial dysfunction [30], and previous studies have shown that patients with exercise-induced ischemia may have more severe endothelial dysfunction than patients without [31]. It can also be speculated that patients with exercise-induced ischemia are more vulnerable to a certain degree of endothelial dysfunction in terms of perfusion disturbances, due to possibly more advanced atherosclerotic disease. In both cases, it might be suggested that the additional impairment of endothelial function caused by insulin resistance, affects perfusion, and thereby oxygen uptake, to a greater extent in patients with exercise-induced ischemia compared to those without.

We realize that the associations presented in the present study are not suitable for implying any causal relationships, and an alternative explanation to the association between insulin resistance and exercise capacity may be that exercise capacity and/or the level of physical activity have modulating effect on insulin sensitivity. Our study subjects, with their combination of type 2 diabetes and CAD, may be habitually sedentary with accompanying low VO_2peak_, and this may contribute to insulin resistance. Physical inactivity has been stated as one of the most important causes of insulin resistance [32].

We did not find any correlation between glucose control and exercise capacity. Although some previous studies have shown an association between glucose control and exercise capacity [10] and that heart rate and pulmonary responses to exercise are reduced in type 2 diabetic patients with poor glucose control [9], our results are in accordance with most studies of type 2 diabetic patients, showing no association between glucose control and exercise capacity [5,33]. This indicates that the presence of CAD in type 2 diabetic patients does not alter these consistent findings.

Most of the significant correlations in the present study are in the range of 0.2 to 0.4, implying that only a minor part of the variance in one parameter is explained by variations in the other. Nevertheless, VO_2peak_ is a complex physiological variable being determined by many different factors, and as it holds important prognostic information, even weaker correlations with this parameter may be of clinical relevance.

### Limitations

The hyperinsulinemic euglycemic glucose clamp technique is the preferred method for measuring insulin resistance [34], but due to practical reasons, the HOMA model was used as a surrogate index of insulin resistance in the present study. This is a well-validated and widely used method [23], although the model has some shortcomings. The HOMA model gives a surrogate index of *hepatic* insulin resistance, and it will not necessarily reflect *peripheral* insulin resistance. Further, the method may not give appropriate results in patients with severely impaired beta-cell function [34].

Although being an inaccurate measure of overweight, BMI was used to adjust for adiposity in the correlations between insulin resistance and exercise capacity. This may have influenced the results.

## Conclusions

Insulin resistance, as estimated by fasting insulin and the HOMA index, was inversely associated with exercise capacity in our study population of patients with type 2 diabetes and CAD, being more pronounced in the subgroup with exercise-induced ischemia. These results indicate that insulin resistance is related to exercise capacity in type 2 diabetic patients with CAD, possibly even more so in patients with exercise-induced ischemia compared to those without.

## Abbreviations

CAD: Coronary artery disease; HOMA-IR: Homeostatic model assessment of insulin resistance; VO2peak: The highest measured oxygen uptake during an incremental exercise test; AT: Anaerobic threshold; AMI: Acute myocardial infarction.

## Competing interests

The authors declare that they have no competing interests.

## Authors’ contributions

RB has been involved in the planning of the study, the recruitment and follow-up of the study participants, the acquisition and interpretation of clinical and laboratory data and in the drafting and intellectual content of the manuscript. EE has contributed to the acquisition and interpretation of the cardiopulmonary exercise test data. IUN has been involved in the recruitment and screening of potential participants, acquisition of clinical and laboratory data and in the clinical follow-up of study patients. HA and IS have contributed to the design of the study, the interpretation of the results and the intellectual content of the manuscript. SS has been involved in drafting of the manuscript, interpretation of the results and the intellectual content. All authors read and approved the final manuscript.
